# Case Report: Diagnostic challenges and therapeutic approaches in cardiac intimal sarcoma: a rare clinical case study

**DOI:** 10.3389/fcvm.2025.1668911

**Published:** 2025-11-04

**Authors:** Chao Ma, Junyi Tang, Yin Xiao, Wenbin Tian, Tao Wang

**Affiliations:** Department of Thoracic and Cardiovascular Surgery, Zhuzhou Central Hospital, Zhuzhou, Hunan, China

**Keywords:** cardiac intimal sarcoma, neoplasms, arterial embolism, breast cancer, misdiagnosis

## Abstract

**Background:**

Primary cardiac tumors are exceedingly rare, with the majority being benign, while malignant tumors are even less common. Cardiac intimal sarcoma represents a rare and aggressive variant of primary cardiac tumors, characterized by its stromal origin and frequent association with genetic abnormalities. They typically present with non-specific symptoms, making early diagnosis difficult.

**Case demonstration:**

A 61-year-old female patient with a medical history notable for multiple neoplasms and surgical interventions is discussed. In 2006, she was diagnosed with uterine fibroids and underwent surgical excision. A decade later, in 2016, she was diagnosed with breast cancer, for which she received surgical treatment followed by radiotherapy. In 2023, the patient developed bilateral lower extremity arterial embolism, and a thrombectomy revealed a mixed thrombus. In 2024, she experienced a recurrence of arterial embolism in the left lower extremity. Following a thrombectomy, the embolus was identified as a myxoma, and subsequent echocardiographic evaluation revealed a mass within the left ventricle. Surgical intervention was performed to excise the left ventricular mass, and the final postoperative pathological examination confirmed the diagnosis of cardiac intimal sarcoma.

**Conclusion:**

Cardiac intimal sarcoma of the left ventricle is an uncommon condition, and the absence of specific symptoms complicates early diagnosis, frequently resulting in misdiagnosis. While chemotherapy and gene-targeted therapies may improve patient outcomes, early and complete surgical resection is crucial for achieving long-term survival in individuals with cardiac intimal sarcoma.

## Background

Primary cardiac tumors are indeed rare, with benign tumors comprising approximately 75% of all cases (1). These tumors frequently present without symptoms and are typically identified incidentally during imaging conducted for other medical conditions. Despite their benign nature, they can occasionally result in significant clinical complications, such as obstruction, embolization, or arrhythmias. The most prevalent benign cardiac tumors include myxomas, lipomas, and rhabdomyomas (2). Myxomas are the most commonly encountered in adults, whereas rhabdomyomas are more prevalent in pediatric populations (3). Malignant cardiac tumors are even rarer, with cardiac sarcomas being the most common type. The scarcity of these tumors contributes to the limited literature and understanding of their optimal management. These malignant tumors often present with nonspecific symptoms, which can lead to delays in diagnosis (4).

Cardiac intimal sarcoma represents a rare and highly aggressive form of primary cardiac tumor, presenting substantial challenges in both diagnosis and treatment. These tumors originate from mesenchymal tissue and are frequently associated with genetic abnormalities, such as MDM2 (Mouse Double Minute 2 homolog) amplification, which can facilitate their identification (5). Despite their infrequency, cardiac intimal sarcomas exhibit marked aggressiveness, often manifesting with non-specific symptoms that can resemble other cardiac conditions, thereby complicating early diagnosis (6). The prognosis for patients with cardiac intimal sarcoma remains generally poor, with survival typically limited to a few months post-diagnosis, attributable to the tumor's aggressive behavior and significant metastatic potential (7).

## Case demonstration

**Figure 1:**
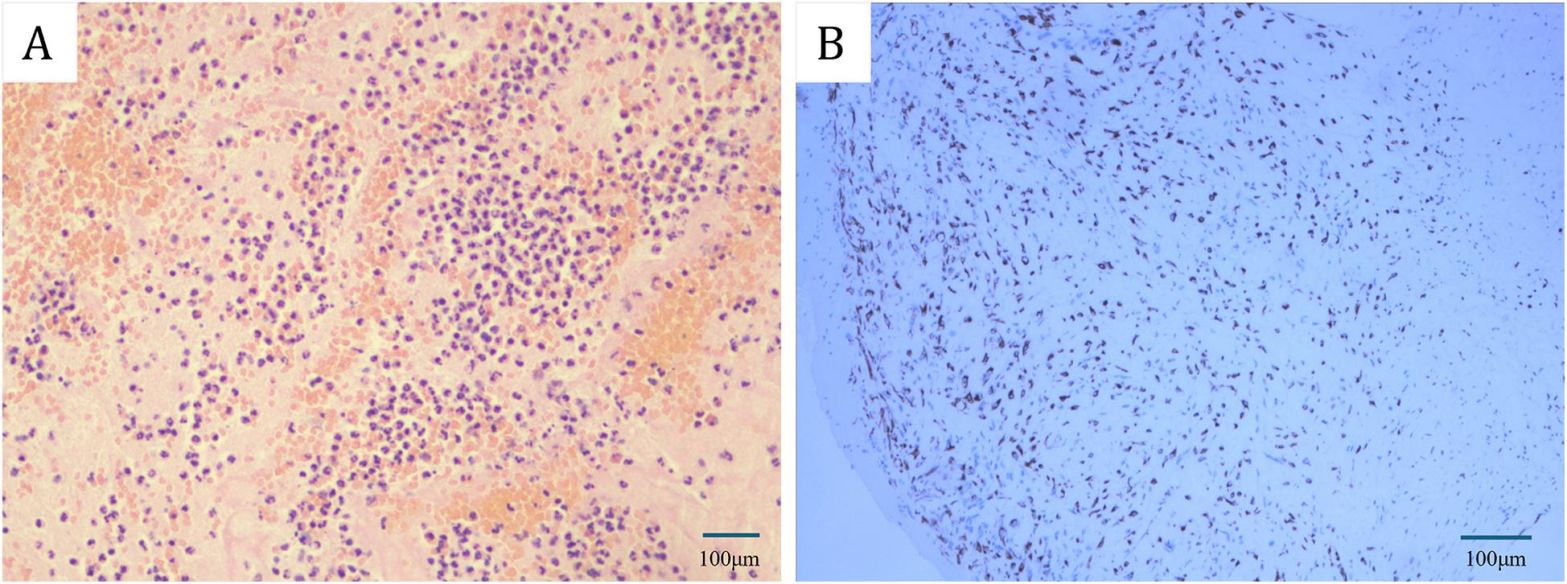
**(A)** Hematoxylin-eosin (HE) staining revealed that the initial emboli in the popliteal artery of the lower extremities were characterized as mixed thrombosis. **(B)** Immunohistochemical staining indicated that the subsequent arterial embolus in the lower extremity was identified as a myxoma. [Bars: **(A)** 100 µm; **(B)** 100 µm].

**Figure 2:**
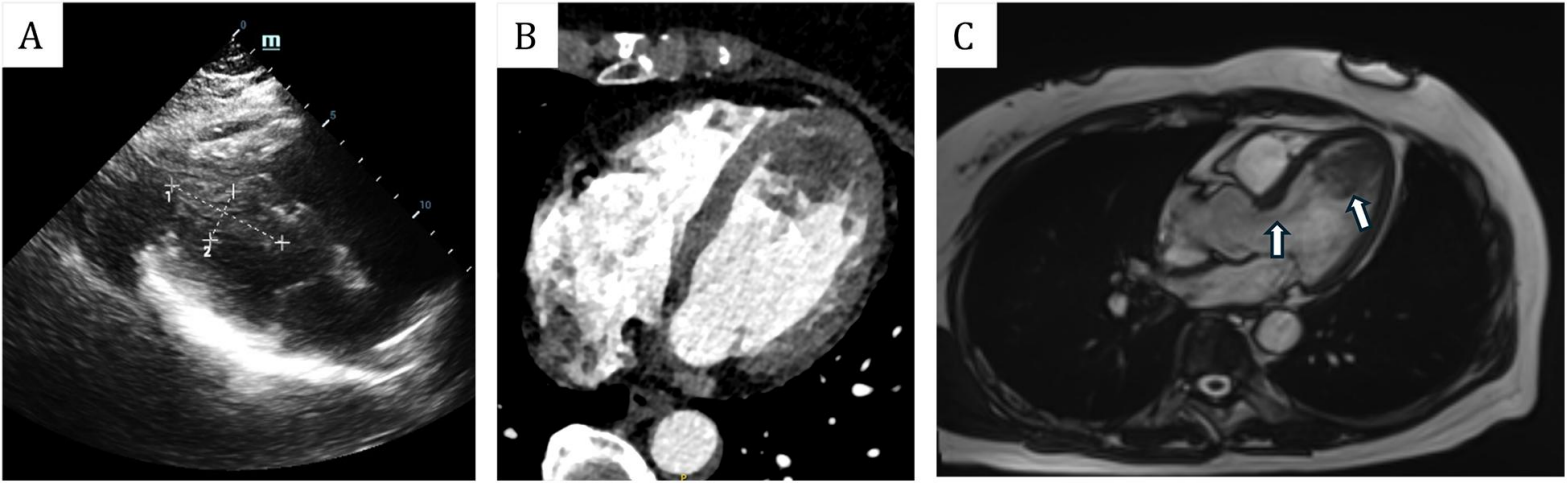
**(A)** Transthoracic echocardiogram showed that the mass was located in the left ventricle, measuring approximately 49 mm × 21 mm. **(B)** Enhanced cardiac computed tomography (CT) revealed an irregularly shaped patchy filling defect within the left ventricle. **(C)** Magnetic resonance imaging (MRI) revealed that the mass was situated in the left ventricle, with its pedicle attached to the ventricular septum. Additionally, flocculent materials were observed extending from the main body of the mass to the aortic valve orifice.

A 61-year-old female patient presents with a medical history characterized by multiple neoplasms and surgical interventions. Initially, in 2006, she underwent a subtotal hysterectomy due to uterine fibroids. A decade later, in 2016, bilateral breast nodules were detected. Ultrasonography revealed a 13 mm × 6 mm nodule in the left breast and a 14 mm × 8 mm nodule in the right breast. Subsequently, she underwent a Mammotome excision of the bilateral breast masses under local anesthesia and B-ultrasound guidance. Postoperative histopathological analysis identified high-grade ductal carcinoma *in situ* in the left breast and fibroadenoma in the right breast. Consequently, a left breast-conserving radical mastectomy was performed. The postoperative specimen revealed no residual carcinoma, and axillary lymph node dissection showed no evidence of metastasis. Three months following the breast cancer surgery, the patient was readmitted for postoperative adjuvant radiotherapy, receiving a cumulative radiotherapy dose of 60 Gy over 30 fractions. Subsequent outpatient follow-ups indicated no recurrence of breast cancer. In 2023, the patient was hospitalized due to bilateral lower extremity numbness. At that time, she reported no symptoms of chest tightness, dyspnea, cough, or expectoration. A computed tomography angiography (CTA) of the lower extremities revealed an occlusion of the right popliteal artery, along with severe stenosis in the right external iliac artery, proximal right femoral artery, and proximal right deep femoral artery. Echocardiography did not detect any cardiac space-occupying lesions at that time. The patient underwent a thrombectomy of the popliteal artery in both lower extremities, postoperative pathological analysis identified the thrombus as a mixed thrombus (Figure 1A). In 2024, the patient was readmitted to the hospital due to weakness in the left lower extremity and reduced skin temperature, without any heart symptoms. CTA of the lower extremities indicated thrombosis and severe stenosis in the left common iliac artery, external iliac artery, and internal iliac artery. Subsequently, she underwent a left iliac artery thrombectomy. Postoperative pathology confirmed a *diagnosis* of myxoma (Figure 1B). Subsequent echocardiographic examination identified a slightly hyperechoic mass in the left ventricle, measuring approximately 49 mm × 21 mm, characterized by well-defined boundaries yet of indeterminate nature. Associated with this mass were flocculent structures extending to the aortic valve orifice, oscillating with the blood flow (Figure 2A). Enhanced cardiac computed tomography (CT) revealed an irregularly shaped patchy filling defect within the left ventricle, indicative of a potential thrombus or neoplasm (Figure 2B). Cardiac magnetic resonance imaging (MRI) further identified a space-occupying lesion in the left ventricle, raising the possibility of a neoplastic lesion, such as a myxoma or vegetation (Figure 2C).

**Figure 3:**
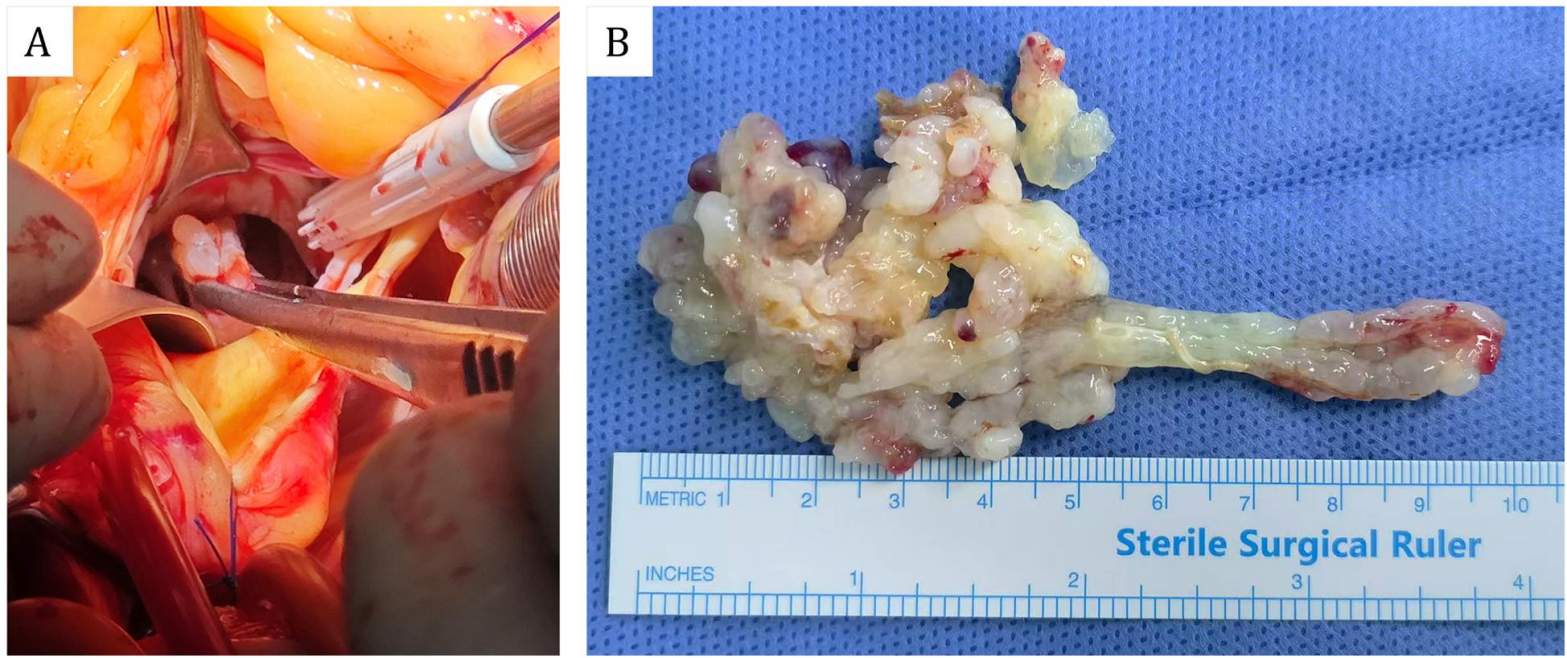
**(A)** Perspective of intraoperative aortic incision. **(B)** Gross specimens of cardiac masses.

**Figure 4:**
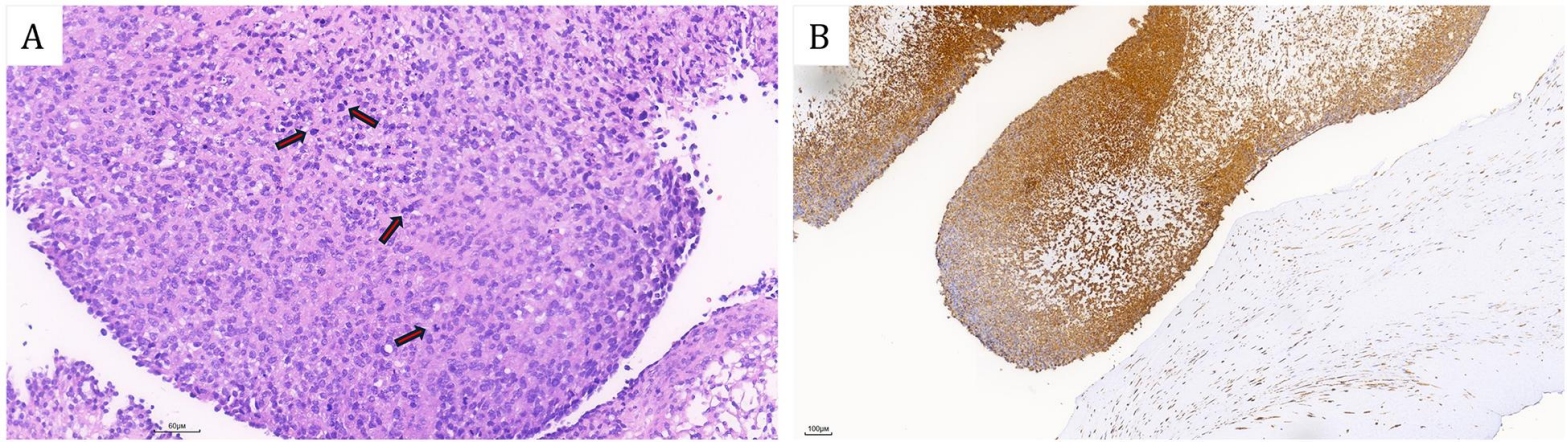
**(A)** Hematoxylin-eosin (HE) stained sections revealed that the tumor cells exhibited a spindle-shaped morphology with atypical protrusions and frequent mitotic activity (Red arrows). Additionally, mucinous changes were observed in the stroma. **(B)** Immunohistochemical revealed diffuse positivity of MDM2. [Bars: **(A)** 60 µm; **(B)** 100 µm].

The patient subsequently resection of a left ventricular tumor, a combined incision was executed at the apex and aorta to excise the left ventricular tumor along with a portion of the interventricular septum tissue to which the pedicle was attached. Intraoperatively, the tumor was identified at the apex of the left ventricle, with its pedicle affixed to the interventricular septum (Figure 3A). The solid mass exhibited a ﬁsh-ﬂesh appearance, characterized by the absence of a capsule, a cauliflower-like morphology, a soft consistency, and a small amount of adherent thrombus and myxoma-like components (Figure 3B). The cardiac cavity was irrigated with a considerable volume of normal saline. The postoperative immunophenotypic analysis demonstrated positive expression for Vimentin, Smooth Muscle Actin (SMA), and Mouse Double Minute 2 homolog (MDM2), with focal positivity for the ETS-related gene (ERG). Negative markers were identified as Myogenin, SOX-10, Thyroid Transcription Factor-1 (TTF-1), Villin, Epithelial Membrane Antigen (EMA), Desmin, Cluster of Differentiation 20 (CD20), and S100 protein (S-100). Partial positivity was noted for Cluster of Differentiation 34 (CD34), and the Ki-67 proliferation index was determined to be 20%. These findings confirmed a diagnosis of cardiac intimal sarcoma (Figure 4). The patient was discharged from the hospital ten days post-operatively. Neither chemotherapy nor immunotherapy was administered following the procedure. Considering the high malignancy and recurrence rate of cardiac intimal sarcoma, the patient underwent monthly color Doppler ultrasound examinations during the first six months post-operation, followed by bi-monthly examinations in the subsequent six months. Additionally, enhanced computed tomography (CT) scans of the heart were conducted at six months and one year post-operatively. After a follow-up period of one year, the patient remains alive with no evidence of cardiac tumor recurrence.

## Discussion

Primary malignant cardiac tumors are exceedingly uncommon, exhibiting an incidence rate ranging from 0.001% to 0.03% among primary cardiac tumors, with sarcomas comprising approximately 95% of malignant cases. An analysis of data from the Surveillance, Epidemiology, and End Results (SEER) database covering the period from 1975 to 2016 revealed an incidence rate for cardiac sarcoma, encompassing all subtypes, of 0.22 per 100,000 person-years (8). Within the category of primary cardiac sarcomas, cardiac intimal sarcoma is the least frequently reported subtype. These tumors are characterized by poorly differentiated spindle-shaped cells and are strongly associated with MDM2 genetic amplification, presenting a significant diagnostic challenge due to their non-distinctive histological features (9). The prognosis for cardiac intimal sarcoma is generally poor, with a mean survival duration ranging from 3 months to 1 year, primarily attributable to the tumor's aggressive nature and its propensity for rapid metastasis.

Cardiac intimal sarcoma is an exceptionally rare and aggressive subtype of primary cardiac tumors. Patients often present with non-specific clinical symptoms, which complicates early diagnosis. Commonly reported symptoms include cough, chest tightness, and dyspnea, frequently leading to initial misdiagnoses as more prevalent conditions, such as pulmonary embolism or cardiac dysfunction. For instance, a documented case involved a patient whose clinical presentation was similar to that of atrial myxoma, highlighting the diagnostic complexities associated with this condition (10). The rarity of cardiac intimal sarcomas further exacerbates diagnostic challenges, as these tumors can mimic other cardiac pathologies in both clinical presentation and imaging findings. In one case, a patient exhibited symptoms such as night sweats, malaise, and dyspnea, initially suspected to be thrombi within the left atrium and ventricle. However, subsequent imaging revealed an intimal sarcoma, underscoring the necessity for comprehensive diagnostic evaluations (11). Cardiac angiosarcoma represents a more prevalent subtype of cardiac sarcoma and requires differentiation from intimal sarcoma. Typically, cardiovascular sarcoma manifests as a brown, multilocular mass with an infiltrative base involving the endocardium. It is frequently associated with necrosis, hemorrhage, and pericardial involvement. Histologically, this sarcoma is characterized by necrotic tissue interspersed among cells, which form irregular vascular lumens, with a predilection for occurrence in the right atrium.

In this case study, the patient exhibited no cardiac symptoms, such as coughing, chest pain, or dyspnea, throughout the progression of the disease. The patient had a medical history significant for multiple bilateral lower extremity embolisms. During the initial episode of lower extremity embolism, echocardiographic evaluation did not reveal any intracardiac space-occupying lesions. Histopathological analysis of the thrombectomy specimen from the lower extremity artery indicated the presence of a mixed thrombus. Although echocardiography at that time did not detect an intracardiac thrombus, the potential cardiac origin of the mixed thrombus could not be excluded. Further transesophageal echocardiogram should have been conducted, as it could have potentially identified preliminary indications of thrombi or neoplasms within the cardiac structure. Six months later, the patient experienced recurrent bilateral lower extremity arterial embolism. Pathological examination of the thrombectomy specimen revealed a myxoma. Concurrently, echocardiography identified a 49 mm × 21 mm slightly hyperechoic mass in the left ventricle, accompanied by a flocculent band oscillating at the aortic valve orifice. Preoperatively, it was hypothesized that the ventricular mass represented a myxoma. Subsequent cardiac computed tomography (CT) revealed an irregularly shaped, patchy filling defect within the left ventricle. Cardiac magnetic resonance imaging (MRI) further identified an irregular intracardiac mass situated at the apex of the left ventricle, with no evidence of myocardial invasion, and possessing a pedicle attached to the ventricular septum. These imaging findings suggest that the mass is amenable to complete surgical resection. The preoperative medical history and examination data were insufficient to determine the nature of the tumor conclusively, however, the preoperative assessments did not indicate any definitive signs of local or distant metastasis, and the patient experienced recurrent cardiogenic arterial embolism, consequently, surgical resection of the cardiac tumor was undertaken. Intraoperatively, the excised specimen was observed to have a pedicle, an irregular shape, a soft texture, and a fish-like appearance. The mass was enveloped in material resembling both myxoma and thrombus, which could plausibly explain the thrombus and myxoma components observed in the arterial embolisms of the lower extremities.

The precise risk factors for cardiac intimal sarcoma remain largely undefined. In this particular case, the patient had previously undergone breast cancer surgery followed by radiotherapy. Although the relationship between radiotherapy and the development of intimal sarcoma is not definitively established, post-radiation sarcoma has been documented in individuals with breast, cervical, and head and neck cancers (12). Nevertheless, certain studies have provided valuable insights. Genetic alterations, notably the amplification of MDM2, have been strongly implicated in cardiac intimal sarcoma. A case report involving a 59-year-old female with cardiac intimal sarcoma and multiple skeletal muscle metastases identified MDM2 oncogene amplification, suggesting its potential involvement in tumor development and metastasis (13). Cardiac intimal sarcoma, a particularly rare form of cardiac sarcoma, presents a challenging relationship with radiotherapy that remains difficult to elucidate. Nonetheless, several studies have documented instances of intimal sarcoma following radiotherapy for tumors located in other regions of the body (5, 14). Consequently, the authors posit that radiotherapy cannot be excluded as a potential factor in the development of endocardial sarcoma in this patient, however, further cases are required to definitively establish this association. Beyond the history of radiotherapy, the patient also has a medical history marked by multiple neoplasms and surgical interventions. This association indicates that prior malignancies and subsequent surgical treatments may contribute to the development of cardiac intimal sarcoma.

Surgical resection remains the cornerstone of treatment for cardiac intimal sarcoma. Due to the rarity and aggressive nature of these tumors, a treatment strategy that emphasizes complete surgical excision to achieve tumor-free margins is crucial, as this approach is associated with improved survival outcomes (15). Furthermore, the integration of multimodal therapies, including chemotherapy and radiotherapy, alongside surgical resection, has demonstrated potential in extending survival and managing recurrent disease. A retrospective analysis of cardiac sarcoma treatments indicated that patients who underwent surgical resection followed by adjunctive therapies experienced reasonable survival rates, even in cases of tumor recurrence or metastasis (16). In our case, no local or distant metastasis was observed in the patient. Additionally, the tumor was attached via a pedicle to the ventricular septum. We expanded the surgical field and successfully achieved complete resection of the tumor within the heart.

## Conclusion

We present a highly uncommon case of cardiac intimal sarcoma located in the left ventricle. The patient exhibited no cardiac symptoms but experienced two consecutive episodes of lower extremity arterial embolism, occurring six months apart. The initial embolism was identified as a mixed thrombus, with no cardiac tumor detected via cardiac color Doppler ultrasound. The subsequent embolism was characterized as a myxoma, which initially led to the erroneous assumption that the cardiac tumor was a myxoma. However, definitive pathological analysis ultimately confirmed the diagnosis of intimal sarcoma. The absence of specific clinical symptoms significantly complicates the early diagnosis of intimal sarcoma, often resulting in its misdiagnosis as other types of cardiac space-occupying lesions. Histopathological examination following surgical resection remains the gold standard for diagnosis. While radiotherapy for certain tumors may act as an inducing factor for intimal sarcoma, genetic alterations also play a crucial role in its pathogenesis. Surgical resection is the primary treatment modality for cardiac intimal sarcoma. Although chemotherapy and gene-targeted therapies can enhance patient prognosis, early and comprehensive surgical resection is essential for ensuring long-term survival in patients with cardiac intimal sarcoma.

## Data Availability

The raw data supporting the conclusions of this article will be made available by the authors, without undue reservation.
